# Adenosine kinase deficiency with neurodevelopemental delay and recurrent hepatic dysfunction: A case report

**DOI:** 10.12715/ard.2014.3.2

**Published:** 2016-07-21

**Authors:** Marjan Shakiba, Fatemeh Mahjoub, Hassan Fazilaty, Fereshteh Rezagholizadeh, Arghavan Shakiba, Maryam Ziadlou, William A. Gahl, Babak Behnam

**Affiliations:** 1 Department of Pediatric Endocrinology, Mofid Children Hospital, Shahid Beheshti University of Medical Sciences (SBMU), Tehran, IR Iran; 2 Department of Pathology, Tehran University of Medical Sciences, Tehran, Iran; 3 Department of Medical Genetics and Molecular Biology, Faculty of Medicine, Iran University of Medical Sciences (IUMS), Tehran, IR Iran; 4 Instituto de Neurociencias CSIC-UMH, San Juan de Alicante, Spain; 5 Medical Student Research Committee (MSRC), Iran University of medical sciences (IUMS), Tehran, Iran; 6 Nutrition Department at International campus, Shahid Sadoughi University of Medical Sciences, Yazd, Iran; 7 Obesity Research Center, Research Institute for Endocrine Sciences, Shahid Beheshti University of Medical Sciences, Tehran, Islamic Republic of Iran; 8 Section on Human Biochemical Genetics, Medical Genetics Branch, National Human Genome Research Institute, NIH, Bethesda, Maryland, USA; 9 NIH Undiagnosed Diseases Program, Common Fund, Office of the Director, NIH, Bethesda, Maryland, USA

## Abstract

Hypermethioninemia may be benign, present as a nonspecific sign of nongenetic conditions such as liver failure and prematurity, or a severe, progressive inborn error of metabolism. Genetic causes of hypermethioninemia include mitochondrial depletion syndromes caused by mutations in the *MPV17* and *DGUOK* genes and deficiencies of cystathionine β-synthase, methionine adenosyltransferase types I and III, glycine N-methyltransferase, S-adenosylhomocysteine hydrolase, citrin, fumarylacetoacetate hydrolase, and adenosine kinase. Here we present a 3-year old girl with a history of poor feeding, irritability, respiratory infections, cholestasis, congenital heart disease, neurodevelopmental delay, hypotonia, sparse hair, facial dysmorphisms, liver dysfunction, severe hypermethioninemia and mild homocystinemia. Genetic analysis of the adenosine kinase (*ADK*) gene revealed a previously unreported variant (c.479–480 GA>TG) resulting in a stop codon (p.E160X) in *ADK*. A methionine-restricted diet normalized the liver function test results and improved her hypotonia.

## Introduction

Hypermethioninemia is a major biochemical sign of certain nongenetic disorders as well as a variety of inborn errors of metabolism. Some genetic diseases exhibit hypermethioninemia secondary to generalized hepatic dysfunction; examples include citrin deficiency, fumaryl acetoacetate hydrolase deficiency and mitochondrial depletion syndromes due to mutations in *MPV17* or *DGUOK.* Other causes of hypermethioninemia involve primary defects in methionine metabolism and the transsulfuration pathway; they include deficiencies of cystathionine β synthase (CBS), methionine adenosyltransferase (MAT) type I and III, glycine N-methyltransferase (GNMT), and S adenosylhomocysteine hydrolase (SAHHD).

Another, unusual cause of hypermethioninemia involves biallelic mutations in the gene for adenosine kinase (ADK), also associated with neurodevelopmental delay, seizures and hepatic dysfunction [5–6]. Here we report an Iranian case of ADK deficiency, with a typical clinical presentation except that the liver disease was more severe and the patient also had a neurologic bladder and red cell macrocytosis.

## Case presentation

This female child of consanguineous parents was born by cesarean section with a birth weight of 2800 g. She was hospitalized the first day of life with jaundice and suspected sepsis, and again admitted to the NICU at 16 days of life with respiratory distress and aspiration pneumonia. During this admission period, a patent ductus arteriosus (PDA) and a large ventricular septal defect (VSD) were detected; she underwent open-heart surgery at four months of age and was treated with digoxin and captopril.

At 8 months, the girl was again hospitalized, this time because of fever, irritability and poor feeding, after an angiography examination. Neurodevelopmental delay and poor head control were noted. On examination, she had mottling, severe hypotonia, and a holosystolic murmur of grade 3/6. Abdominal ultrasonography revealed hepatomegaly with a homogenous echo pattern, a small amount of free fluid in the abdomen, and questionable intussusception. Elevated transaminase levels and macrocytosis were prominent findings. Laboratory results are shown in Table 1. The patient was treated with ceftriaxone, discharged on co-amoxiclav, and referred for follow-up liver function tests.

After another 3 months, the patient was again admitted with fever, productive cough, irritability and poor feeding She had a seborrheic rash on the scalp and an erythematous rash on the abdomen, as well as buccal candidiasis, a systolic murmur, and hypotonia.

The serum aspartate aminotransferase (SGOT) and alanine aminotransferase (SGPT) values were 300 and 400 IU/L, respectively. Gamma-glutamyl transferase (GGT) was 146 IU/L, and alpha-1 antitrypsin (A1AT) was within normal limits. Other laboratory results are listed in Table 1. Virology tests for EBV, CMV, HAV, HBV, HCV and HIV were negative. On metabolic consultation, her weight, height and head circumference were in the 5^th^ percentile for age. Dysmorphisms included frontal bossing, hypertelorism, a palpebral fissure slant, depressed nasal bridge and flat zygoma, sparse, thin blond hair, with no similarity to her parents, and severely carious teeth.

Galactose-1-phosphate uridyltransferase (GALT) activity, and acylcarnitine profiles, and urine succinylacetone were normal. Plasma amino acid analysis revealed severe hypermethioninemia; plasma homocysteine was also elevated at 29 mg/dl (reference range: 5–16).

The patient was treated with vitamin B6 (360 mg), betaine (200 mg/kg/d), vitamin B12 (1000 μg), folate (2 mg), and methionine restriction. At follow-up, the patient’s neurodevelopmental condition was much improved, and liver function had nearly returned to normal.

At the age of 18 months, the patient was again admitted to hospital with fever and gastroenteritis. She was icteric, her SGOT was 5070 IU/L, SGPT 4050 IU/L, total bilirubin 12 mg/dl, direct bilirubin 6.2mg/dl, prothrombin time [PT] 23 s, international normalized ratio [INR] 2.7, and partial thromboplastin time [PTT] 63 s. Urinary retention was detected. Urine analysis and culture revealed a *Klebsiella* urinary tract infection. A voiding cystourethrogram (VCUG) showed a neurogenic bladder.

Liver crises were associated with infection and other stressors such as surgery. Hypermethioninemia during an intercurrent illness was recorded on several occasions (1140, 1304, 1440 μmol/L). Methionine levels were also high during stable phases (985, 809, 1200 μmol/L).

A methionine-restricted diet improved the liver function tests better than did the administration of B6, B12, betaine, or folic acid. The diet limited protein to 2 g/kg/day, half from metabolic formula (without methionine, valine, threonine, and isoleucine) and half as natural protein from regular sources such as breast milk, infant formula, and table foods. High protein foods such as meat, egg, poultry, dairy products, nuts, legume, and chocolate were omitted from her diet. The amount of methionine in the diet was 17–20 mg/kg/day. A specific metabolic low protein book [7] gave the family information on the protein and calorie contents of various foods; on average, 1 gram of protein contains 20 mg of methionine. With rigid dietary control, the girl’s methionine has been within normal limits for the past 1.5 years.

**Figure 1:**
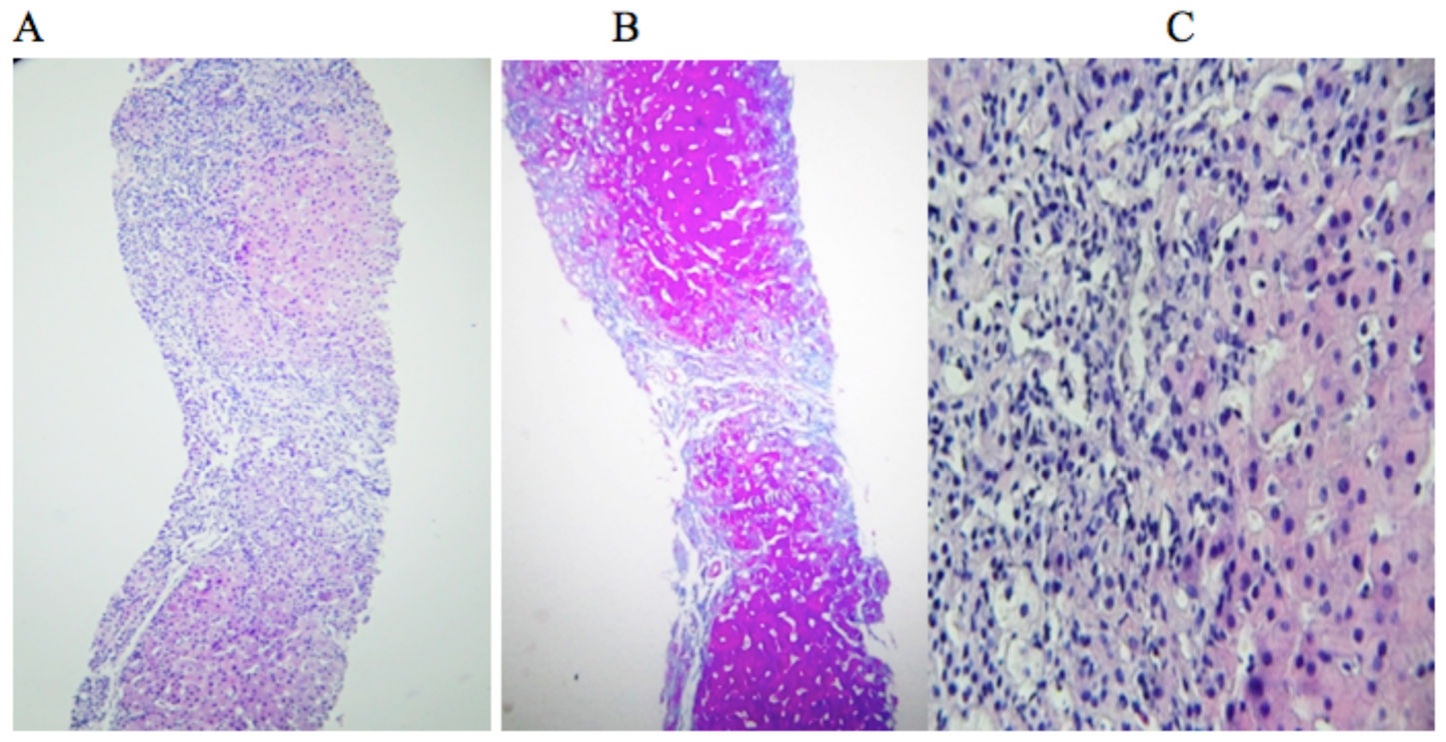
Histopathology examination of hepatocytes. A: Liver tissue with expansion of the portal areas by infiltration of inflammatory cells. B: Trichrome stain showing bands of fibrous tissue forming portal-portal bridges (stage 4). Hepatocytes show anisonucleosis and double nuclei. Foci of confluent necrosis were seen. C: Portal area with infiltration of lymphocytes, some eosinophils, and few neutrophils resulting in interface. Foci of the infiltration of intralobular inflammatory cells were seen and the bile duct is unremarkable.

Since the clinical presentation of the patient’s disease was incompatible with classic homocystinuria, and the cause of the hypermethioninemia was unknown, a liver biopsy was performed. The tissue displayed expansion of the portal areas forming portal-portal bridges (Stage 4), and moderate infiltration of lymphomononuclear cells and some eosinophils in the abovementioned areas. Foci of infiltrating intralobular inflammatory cells were seen, and hepatocytes showed anisonucleosis and double nuclei. Foci of confluent necrosis were seen; the bile duct was unremarkable (Figure 1).

Measurements of S-adenosyl methionine (SAM), S-adenosyl homocysteine (SAH) and urine adenosine were not available, but the patient’s clinical presentation matched closely with ADK deficiency, and genetic testing was performed.

### ADK gene analysis

Genomic DNA was extracted from peripheral blood leukocytes and all coding regions of the *ADK* gene, including intron-exon boundaries, were PCR amplified using primers binding within adjacent introns of each exon. Primer sequences are given in Table 2. Single strand sequencing was performed using the standard ABI3730 system (Applied Biosystems, Macrogen, South Korea). Sequencing results were analyzed using Chromas software (Technelysium Pty Ltd., Brisbane, Australia; version 2.4.1) and sequences were aligned to the published template (accession no. NM_001123) using Clustal Omega software (EMBL-EBI, Cambridge, UK).

**Figure 2:**
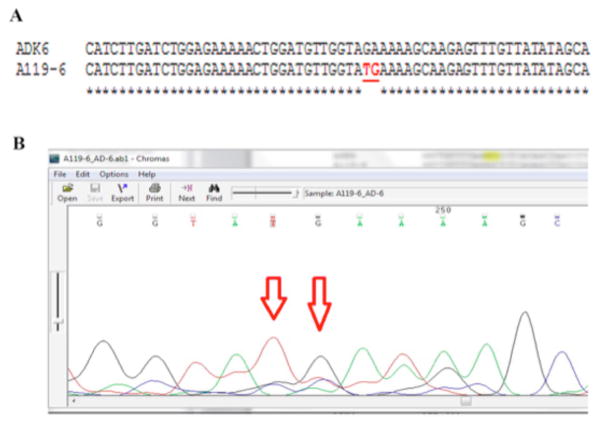
Sequencing result for ADK exon 6. A: Patient’s sequence aligned to published template (NM_001123) using Clustal Omega software. B: Corresponding chromatogram (Chromas software version 2.4.1) for the region containing alterations. Red arrows show the altered nucleotides.

Observed alterations in gene sequences were checked against published mutations and polymorphisms, and for conservation across species. Two adjacent substitutions in positions 479 and 480 were seen in exon 6; these homozygous changes led to stop codons (c.479–480 GA>TG; p.E160X), confirming a diagnosis of ADK deficiency. Aligned sequences and a corresponding chromatogram are shown in Figures 2A and 2B, respectively.

## Discussion

If mild, hypermethioninemia can be benign. Severe hypermethioninemia, however, can reflect a progressive inborn error of metabolism or a nongenetic condition such as liver failure or prematurity. Deficiencies in four enzymes - methionine S-adenosyltransferase (MAT I/III), glycine N-methyltransferase (GMT), S-adenosylhomocysteine hydrolase (SAHH) and cystathionine β-synthase (CBS) - have been associated with hypermethioninemia, each leading to a disruption in the transsulfuration pathway.

Cystathionine β synthase deficiency presents with neurologic problems, marfanoid features and lens dislocation as the major manifestation. Patients with methionine adenosyltransferase deficiency usually have no clinical presentation except for an unpleasant odor. Hepatomegaly and slightly elevated transminases are the main manifestations of glycine methyltransferase deficiency [1]. Rare cases of S-adenosylhomocysteine hydrolase deficiency have been reported with psychomotor delay and muscular hypotonia and chronic active hepatitis[2]. Fumarylacetoacetate hydrolase deficiency, results in type 1 tyrosinemia with hepatorenal involvement, and a high level of succinylacetone in blood plasma and urine. Patients with citrin deficiency have cholestasis in infancy and neurologic and psychiatric manifestations in adulthood [3]. Some reports have linked hypermethioninemia and hepatic abnormality to mitochondrial depletion syndrome caused by mutations in the *MPV17* and *DGUOK* genes [4].

Recent studies have supported a close relationship between the adenosyl and methionine cycles. During the embryonic period, adenosine degradation to inosine is catalyzed by adenosine deaminase. However, after birth, adenosine is largely metabolized by its conversion to adenosine monophosphate (AMP) by adenosine kinase [2, 8]. The mechanisms through which adenosine kinase deficiency can be pathogenic include cellular adenosine toxicity and detrimental effects of decreased AMP levels on cellular and mitochondrial functions. In addition, adenosine can inhibit the immune response and contribute to delayed neurotransmission via abnormal hormone secretion [5–6], and adenosine is also a component of many vital enzymes [5]. Accumulation of adenosine also reverses the SAHH reaction, leading to high levels of adenosyl homocysteine, which impedes movement through the methionine cycle. Since adenosylmethionine acts as a methyl group donor for many substrates, ADK deficiency interrupts methyltransferase processes in many pathways [9].

The incidence of adenosine kinase deficiency remains unknown. There are 20 patients known to have ADK deficiency, but the disease may be underdiagnosed because of limited available data and variable manifestations [6].

This case report introduces a Persian patient presenting with neurodevelopmental delay, severe hypotonia, direct hyperbilirubinemia, signs of liver dysfunction, a syndromic face, sparse hair, congenital heart defects, and susceptibility to infection and neurogenic bladder. She had high levels of liver transaminase and prolonged PT, especially during intercurrent illness and infection. Hypermethioninemia (more than 30 times the normal level) was a permanent biochemical feature of her disease. As in previous reports, hyperhomocystinemia (29μmol/L) was present. Macrocytosis was frequently found in the patient’s cell blood count (CBC) reports, but hypoglycemia was not a prominent manifestation in this case.

We approached this patient considering liver dysfunction as the basis of her hypermethioninemia, since her liver function tests were strikingly elevated. However, her high level of plasma methionine is not a routine finding in generalized liver disease, so we considered disorders specific to the transsulfuration pathway. Deficiencies in MAT and CBS deficiency do not cause liver abnormality. A few cases of GMT deficiency report mild involvement of the liver, but not with associated psychomotor delay or hypotonia, and with less severe liver dysfunction compared to our case. SAHH deficiency, mitochondrial DNA depletion syndrome and ADK deficiency were the main differential diagnoses in this case. Several cases of SAHH deficiency have been reported with similar clinical problems including hypotonia, psychomotor delay and liver dysfunction [10].

Two cases of hypermethioninemia had mitochondrial depletion. The first case was a male child with normal cognitive and early motor milestones, who presented with low appetite, weight loss, mild transaminase elevation, severe hypermethioninemia, and hepatocellular carcinoma. After liver transplantation, he proceeded to progressive liver failure, renal failure, muscle wasting, seizures and loss of muscle tone. Molecular studies revealed MPV17 deficiency and mtDNA depletion. The second case was a female child born with hypotonia, hyporeflexia, and progressive liver and renal failure. The patient was homozygous for a deletion in the gene encoding DGUOK [4].

Six cases of ADK deficiency have been reported; all had severe hypotonia, profound psychomotor delay, macrocephalus, frontal bossing, hypertelorism, sparse language, and seizures. Laboratory analyses revealed hypermethioninemia, normal or high levels of homocystinuria, direct hyperbilirubinemia, and elevated transaminase levels. In one case, liver biopsy revealed slight portal fibrosis and steatosis. Three cases had congenital heart disease, and two were deaf. ADK deficiency was confirmed using whole exon sequencing in these cases [5]. Staufner reported 11 patients (age range: 1.9 to 29 years old) from eight families affected by ADK deficiency. In these cases, clinical manifestations were different, comprising hypoglycemia, epilepsy, liver dysfunction, failure to thrive, cardiac defects, frontal bossing, hypertelorism, and megaloblastic anemia. Elevated S-adenosylmethionine and S-adenosylhomocysteine levels were a constant biochemical finding in these patients, even in the presence of normal plasma methionine levels [6].

In this study, ADK deficiency was suspected and confirmed via sequencing of the *ADK* gene. This revealed a previously unreported homozygous alteration in two adjacent nucleotides; this frameshift resulted in a stop codon at position 160, likely leading, to truncation of the ADK protein.

Our patient had a history of susceptibility to infections such as pneumonia and sepsis-like presentations without a positive culture for microorganisms, buccal candidiasis, viral respiratory infections, or gastroenteritis. This may have occurred because of an impaired immune status; adenosine can inhibit the immune response and contribute to abnormally delayed neurotransmission of hormone secretion [5–6]

Neurogenic bladder, not reported in previous cases, was a problem in our patient. We cannot definitively correlate this finding with ADK deficiency, but it could be a result of decreased AMP and ATP levels since purinergic signaling contributes to nervous system activities, including neuroprotection, central control of autonomic functions, control of vessel tone and angiogenesis, muscle tone, and mechanosensor transduction [11]. No macrocephaly was observed in our patient, but her head circumference increased from the 5^th^ to the 25^th^ percentile for age during the follow-up. Methionine restriction had variable efficacy in the patients reported by Staufner [6]; our case had a significant response to methionine restriction, with improvement in neurodevelopemental condition, growth, cessation of admissions, and normalization of liver transaminase and methionine values.

## Conclusions

ADK deficiency must be considered when the symptoms including cholestasis, liver dysfunction and psychomotor delay or regression are diagnosed, especially in the presence of hypermethioninemia. We recommend a methionine-restricted diet in the treatment of ADK deficiency.

## Figures and Tables

**Table 1:** Patient’s test results at first and second admissions to our hospital

Laboratory test	First admission	Second admission	Reference range
WBC	7,600 (P: 49%, L: 46%)	15,000 (P: 40%, L: 59%)	(6–17.5)×1000
RBC	3.6 ×10**^6^**	4.54×10**^6^**	(3.70 – 5.30)×10**^6^**
HB (HCT)	9.5 (32)	12.6 (38.9)	11.5–15 (35–45)
MCV	92	92	77–86
PLT	271,000	223,000	150,000–400,000
ESR	1%	3%	< 10%
VBG	Normal	Normal	Normal
BS	86	58	60–140 (mg/dl)
BUN	11	18	5–18 (mg/dl)
Cr	0.4	0.3	0.2–0.6 (mg/dl)
Uric acid	-	3.9	1.7–5.8 (mg/dl)
TG	117	-	50–140 (mg/dl)
Cholesterol	137	-	50–170 (mg/dl)
PT	32, 13.5	12.8	9.5–13.5
INR	2.56, 1.07	-	0.8–1.2
PTT	36	-	28–38
Total protein	5.2	-	6.1–7.9 (g/L)
Albumin	3.7	-	3.5–5 (g/L)
SGOT	87, 3240, 468	3240, 159, 115, 1365, 399, 322	15–45 (IU/L)
SGPT	272, 3320, 2080	3320, 254, 189, 965, 794, 307	15–45 (IU/L)
Gamma-GT	-	147	5–32 (IU/L)
Bil (total)	7, 4.4	7.9, 2.8, 1.69	0.2–1.2 (mg/dl)
Bil (direct)	1.5, 0.4	5.6, 2, 1.34	0.1–0.4 (mg/dl)
ALP	736	831	200–1200 (IU/L)
Ammonia	-	62.1	15–58.8 (mg/dl)
Lactate	-	11.8	7–20 (mg/dl)
Homocysteine	8	29	5–16 (μmol/L)
CPK	-	123	5–130 (IU/l)
Ferritin	>1000	1019	7–140 (ng/dl)
Plasma amino acids HPLC			
Tyrosine	-	89	10–145(μmol/L)
Methionine	-	1322	12–40 (μmol/L)

Urine carbohydrate and urine amino acid chromatographies were both checked and showed a normal pattern at first admission.

CBC, cell blood count; WBC, white blood cell count; P, polymorphonuclear; L, Lymphocyte; HB, hemoglobin; HCT, hematocrit; MCV, mean cell volume; PLT, platelet; VBG: venous blood gases; BUN, blood urea nitrogen; Cr, creatinine; TG, triglycerides; PT, prothrombin time; INR, international normalized ratio; PTT, partial thromboplastin time; SGOT, serum glutamic oxaloacetic transaminase; SGPT, serum glutamic pyruvic transaminase; Gamma GT, gamma-glutamyltranspeptidase; BIL, bilirubin; ALP, alkaline phosphatase; CPK, creatine phosphokinase; HPLC, high performance liquid chromatography.

**Table 2:** *ADK* gene-specific primers

Primer symbols	Sequences
ADK-1F	GTGAGCTGGCACGAGACAC
ADK-1R	ATGAAAAGTGCGGAGGGAAC
ADK-2F	TCTGCAACCTTGACACCATC
ADK-2R	TTCCCAAGGAAAACTGTACTCAG
ADK-3F	CCTTTCAGTTCCTGGAGTGG
ADK-3R	TGATCCACCGGAGTAAGACC
ADK-4F	TGACCTCCATTTGGCAATC
ADK-4R	TCCCAATTCAAATGAACAAAAC
ADK-5F	TTGAAATCCCATTCATAACAGC
ADK-5R	CAAGGCATTGAGCAAGCTATC
ADK-6F	CATAGATGCCTCAGAAAGTTCTC
ADK-6R	GGTTGGCAAGCACCTATG
ADK-7F	CTGAGAGTGACTGTGGAGATGG
ADK-7R	TGTGGTCATAGTAACAGGACAGG
ADK-8F	TGTGTTGACATTAGGCTGCC
ADK-8R	ATGACGACTGCCAAGTTTCC
ADK-9F	GGGTTCCTTGGAAGTCACTG
ADK-9R	TTGATTGACAAAATCCCCAAG
ADK-10F	TACTTGCAGATGATTTTGCACC
ADK-10R	CATTTAAGCCTGAAGGGCTATG
ADK-11F	ATATTGGTCTGACCCAATATGAC
ADK-11R	TGACAAGTTTTTGTTTGTGTCC
